# A complementary systems account of word learning: neural and behavioural evidence

**DOI:** 10.1098/rstb.2009.0111

**Published:** 2009-12-27

**Authors:** Matthew H. Davis, M. Gareth Gaskell

**Affiliations:** 1 MRC Cognition and Brain Sciences Unit, Cambridge, UK; 2 Department of Psychology, University of York, York, UK

**Keywords:** pseudoword, meta-analysis, hippocampus, speech, sleep, memory

## Abstract

In this paper we present a novel theory of the cognitive and neural processes by which adults learn new spoken words. This proposal builds on neurocomputational accounts of lexical processing and spoken word recognition and complementary learning systems (CLS) models of memory. We review evidence from behavioural studies of word learning that, consistent with the CLS account, show two stages of lexical acquisition: rapid initial familiarization followed by slow lexical consolidation. These stages map broadly onto two systems involved in different aspects of word learning: (i) rapid, initial acquisition supported by medial temporal and hippocampal learning, (ii) slower neocortical learning achieved by offline consolidation of previously acquired information. We review behavioural and neuroscientific evidence consistent with this account, including a meta-analysis of PET and functional Magnetic Resonance Imaging (fMRI) studies that contrast responses to spoken words and pseudowords. From this meta-analysis we derive predictions for the location and direction of cortical response changes following familiarization with pseudowords. This allows us to assess evidence for learning-induced changes that convert pseudoword responses into real word responses. Results provide unique support for the CLS account since hippocampal responses change during initial learning, whereas cortical responses to pseudowords only become word-like if overnight consolidation follows initial learning.

Recent advances in information technology have led to significant changes in both the activities of daily life and the words that we use to describe those activities. Many of us ‘google’ information on the Internet, looking for articles and commentary from influential ‘blog’ writers or ‘bloggers’; activities and words that have become increasingly common in the last decade. A search of the Google index in January 2001 (via www.google.com/search2001.html) yields 76 000 pages containing the word ‘blog’, whereas the same search in January 2009 reveals 3.2 billion hits. There are more Google hits for ‘blog’ than for ‘tv’ (2.3 billion) and ‘science’ (0.78 billion). These observations illustrate how a contraction of the phrase ‘weblog’ first coined in May 1999 (see Origins of ‘Blog’ and ‘Blogger’, American Dialect Society Mailing List, 20 April 2008) has gone from being an obscure piece of technological jargon to a familiar word for many speakers of English.

In this paper we examine the neurocomputational processes by which new words such as ‘blog’ come to achieve their status as familiar and meaningful units stored in the brains of language users. We propose that: (i) Isolated representations of new words are initially encoded like other novel experiences as ‘episodic’ memories of their first occurrences. These representations are supported by medial temporal lobe memory systems, functionally and neurally distinct from (ii) the neo-cortical representations that support long-term retention of words. Stable cortical representations are derived from multiple encounters with new words by consolidation processes that abstract away from the episodic representations that encode specific occurrences of novel words. Thus two distinct stages of learning and representation (initial episodic and subsequent lexical) are associated with the acquisition of new words.

This account of word learning directly parallels existing dual-systems accounts of memory processes in other domains. The goal of the current paper is therefore to develop a cognitive and neuroscientific account of word learning that reflects the computational constraints proposed by general accounts of memory processes. By integrating this framework with the specific computational demands of the perception of spoken language we can make detailed behavioural and neural predictions concerning processes involved in word learning. While the majority of this paper is focused on the acquisition of novel spoken rather than written words, our goal in developing this account is to achieve a broad coverage of phenomena that are relevant for word learning in general.

The outline of this paper is as follows. We first explore the complementary learning systems (CLS) model (McClelland *et al*. 1995), which partitions memory into hippocampal and neocortical components. These two systems contribute differentially to initial acquisition of episodic memories, and subsequent long-term retention, with transfer of information between these systems through offline consolidation. In the second section, we consider predictions from CLS accounts concerning the acquisition of new spoken words. These predictions are framed within the computational context of the Distributed Cohort Model of spoken word recognition (Gaskell & Marslen-Wilson 1997), and neuroanatomical constraints suggested by dorsal and ventral pathway accounts of spoken language processing (Scott & Johnsrude 2003; Hickok & Poeppel 2004; Davis & Johnsrude 2007). Unifying these two aspects into a single, neurocomputational account of word learning requires that we: (i) specify the functional contribution of neocortical systems that retain long-term representations of spoken words, and (ii) explain how hippocampal/medial temporal memory systems interface with neocortical systems during acquisition. We will propose specific computational signatures of processes supported by rapid, hippocampal learning as distinct from slower neocortical learning mechanisms. Of particular interest, here, is the proposal that learning involves overnight, sleep-associated consolidation processes to mediate between fast-learning hippocampal and slow-learning neocortical systems. We will also derive specific predictions for behavioural differences between newly learned and consolidated novel words.

Next we will review behavioural, neuropsychological and functional imaging evidence relating to this dual-process account. We begin by assessing behavioural evidence for two distinct stages of word learning. A particular focus of recent behavioural research has been to explore cognitive processes such as lexical competition that are unique to familiar words. Consistent with the CLS accounts we review evidence that newly learned words only become effective lexical competitors after offline consolidation. The next two sections review neuropsychological and functional imaging evidence for the involvement of two distinct neural systems in the medial temporal lobe and neocortex in word learning. We examine the evidence of impaired word learning in amnesic individuals with acquired or developmental injuries to the hippocampus, for whom word learning should be severely impaired according to the CLS account. Evidence that the medial temporal lobe contributes to initial word learning is also provided by functional imaging studies in which participants are scanned while learning new words. We then review evidence for cortical involvement in word recognition and learning. A particular focus for the CLS account is evidence from functional brain imaging studies in which neural responses are measured at different stages during the acquisition of novel words. Interpretation of these findings requires background information on the cortical systems that respond differently to familiar and unfamiliar spoken words. We therefore report a meta-analysis of functional imaging studies that compare words and pseudowords and test predictions derived from this meta-analysis for changes in neocortical responses to pseudowords following initial learning and consolidation. Finally we set out some outstanding questions and directions for future research.

## 1. The cls model

The CLS model has much in common with other accounts of memory that invoke distinct learning systems specialized for different types of memory (e.g. procedural versus declarative, Squire 1992; semantic versus episodic, Tulving 1972). However, the CLS account goes beyond these traditional descriptions by considering the computational properties of neural networks that learn from experience. An important distinction for network models is whether learned information is stored in independent, sparse codes (in which distinct memories have little or no overlap in their neural representations), or as overlapping, distributed representations that capture the similarity structure of specific domains of knowledge. The CLS account proposes two distinct memory systems for unique, context-specific representations (episodes), or knowledge that must be generalized beyond the specific context in which it is learned (semantic representations). These two forms of learning are best achieved by computational mechanisms that produce sparser, more independent representations and more overlapping, distributed representations, respectively.

As well as providing a computational basis for these distinct forms of memory, the CLS account also addresses many decades of research in neuropsychology and behavioural neuroscience aimed at understanding the specific contributions of the hippocampus and medial temporal lobe systems that are impaired in amnesia (e.g. Scoville & Milner 1957; O'Keefe & Nadel 1978; Squire 1992), and the neocortical memory systems that support the residual learning abilities of amnesic patients (e.g. Milner 1972). The CLS account proposes that sparse representations are rapidly and efficiently learned by the hippocampus and medial temporal lobe systems whereas overlapping, distributed representations are more slowly learned by the neocortex. Hence, amnesia caused by lesions to the hippocampus results in damage to learning mechanisms that are required to encode experiences rapidly into memory. Conversely, those aspects of memory function that are preserved in amnesic patients (such as repetition priming or motor learning), arise from the operation of neocortical learning processes independent of the hippocampus.

### (a) Computational properties of the CLS model

As we have described, the computational properties of two distinct forms of connectionist learning algorithm are central to the CLS account. The motivation for these two forms of learning is a consideration of the strengths and limitations of neural networks taught using gradient descent learning algorithms. The parallel distributed processing (PDP, Rumelhart & McClelland 1986) approach to connectionist modelling of psychological processes commonly makes use of hidden units to mediate between input and output representations. These three-layer networks are trained to map from input to output by implementing gradual weight changes in the connections, using an algorithm such as backpropagation (Rumelhart *et al*. 1986). Such models have many attractive features, including the ability to generalize from trained mappings to novel items and performance that degrades gracefully in the face of simulated lesions (see Rumelhart & McClelland 1986 for discussion).

However, robust performance and generalization has an associated cost since the use of gradual weight changes means that novel mappings can only be incorporated into a trained network slowly over the course of many presentations, and then only if the novel mapping is interleaved with instances of existing mappings. As McClosky & Cohen (1989) demonstrated, a dichotomous shift in the training patterns can lead to ‘catastrophic interference’, in which learning of new mappings eliminates previously learned mappings (see French 1999 for discussion). McCloskey and Cohen illustrated this with simulations of the AB–AC memory paradigm (Barnes & Underwood 1959), in which participants first learn associations between word pairs (A–B) and are then required to learn associations between the first of the original words and new words (A–C pairs). Learning of new associations in humans typically produces a moderate reduction in recall of A–B pairings, but in the connectionist networks, the effect of the new mappings was to essentially erase the network's memory for the original mapping. This aspect of the network's performance is perhaps unsurprising given that the distributed architecture encourages all mappings to rely on the same set of connection weights—what is needed is a means of keeping newly learned mappings separate from the existing network on a temporary basis until new information can be properly integrated.

The CLS model (McClelland *et al*. 1995; O'Reilly & Rudy 2000; Norman & O'Reilly 2003) provides a potential solution to this *stability–plasticity dilemma* (Carpenter & Grossberg 1988). McClelland *et al*. described a dual-memory system model in which the main (neocortical) memory system operates using a learning algorithm producing distributed representations, as described above. This network has the ability to retain stable memories for long periods, despite changes in the form of the input (i.e. to generalize), and the structure of the network (i.e. robustness to damage). A second, hippocampal, system provides plasticity and can acquire new episodes without interference from previously or subsequently learned knowledge. This network is distinguished by its use of more sparse or near-localist representations, allowing representational independence and a means for swift learning of new patterns without overwriting existing knowledge. These rapidly learned representations in the hippocampal system can then be used to support slower, interleaved learning within the cortical system.

The CLS model has been applied to a broad range of memory phenomena such as the recollection/familiarity distinction in human recognition memory (e.g. Norman & O'Reilly 2003) and discrimination and transitive inference tasks in rats (Frank *et al*. 2003; Atallah *et al*. 2008). The details of the CLS model have been fleshed out (e.g. O'Reilly & Rudy 2000; Norman & O'Reilly 2003), particularly for the hippocampal model and its interaction with other parts of the anterior and medial temporal lobe, which are important for acquisition and representation of unique instances. Recent instantiations of the CLS model also incorporate more neurobiologically realistic learning rules that can be implemented using only local connections, and weight-update schemes (O'Reilly 1996). Nonetheless, the fundamental distinction between neocortical and hippocampal systems has not been substantially altered in recent descriptions and simulations.

In terms of interaction between the two systems, McClelland *et al*. (1995) suggested that a process of offline reinstatement of hippocampal memories drives further learning in the neocortex and a gradual reduction in the dependence of the memory on the hippocampus. At the same time, hippocampal memory traces were assumed to decay, either passively or through interference from newly instantiated hippocampal memories. In combination, these properties are invoked as an explanation of hippocampal amnesia. The reliance on the hippocampal network for the initial encoding and reinstantiation of episodic memory explains why amnesics are generally unable to retain such memories. On the other hand, memories that are formed gradually over the course of many different exposures, such as procedural skills, are more amenable to learning via the neocortical route despite the absence of short-term hippocampal storage. Once again, this fits with the classic description of learning abilities in amnesics (e.g. Squire 1992), although interestingly evidence of learning may not be restricted to non-declarative knowledge if the test of retention is chosen carefully. The CLS model does not involve any form of ‘gating’ in which memories are directed to the relevant system according to their type. Thus, new declarative knowledge should be processed by the neocortical route as well as the hippocampal route even though the effect of the neocortical exposure may be limited, given the gradual nature of learning in that system.

Bayley *et al*. (2008) tested the prediction that some limited knowledge can be supported solely through neo-cortical learning in two amnesics with near complete damage to the hippocampus and associated medial temporal lobe structures. Previous research had suggested that these amnesics showed no memory of post-onset facts and faces in conventional tests of recognition or recall (Bayley & Squire 2005). However, in tests which permit participants to base their responses on feelings of familiarity (e.g. picking a famous face from non-famous foils), both amnesics showed evidence of some declarative knowledge acquired post-lesion although both were well below control performance. This result fits with the idea that the neocortex can incorporate new declarative information to some extent, even when interactions with the hippocampal system are unavailable (see also Duff *et al*. 2006). We will return to this question in §4 where we consider the impact of hippocampal lesions on word learning.

A final crucial prediction of the CLS account relates to retrograde amnesia. Hippocampal amnesics typically reveal impaired performance on recall of memories prior to the onset of brain damage, with the severity of the impairment reducing as the time between memory establishment and amnesia onset increases (Ribot 1882; Kapur & Brooks 1999). Similar effects can be found in the laboratory by lesioning the hippocampus in rats at different times following the formation of a new association (Takehara *et al*. 2003; Tse *et al*. 2007). These findings can be explained by the CLS model in terms of the gradual transfer of hippocampal knowledge to the neocortex, and provides a measure of the duration of the hippocampal–neocortical transfer process (several years in humans, several weeks in rats—though faster consolidation may also be possible in highly schematized domains, Tse *et al*. 2007).

### (b) Sleep and the CLS model

Reinstatement of hippocampal memories in order to strengthen neocortical representation was considered by McClelland *et al*. (1995, p. 424) to involve ‘active rehearsal, reminiscence, and other inactive states including sleep’. The potential involvement of sleep in the transfer process was motivated by research on hippocampal place cells in rats. Wilson & McNaughton (1994); see also Skaggs & McNaughton (1996) correlated firing rates of hippocampal cells responding to particular locations during activity with the firing rates of those same cells in subsequent slow-wave sleep (SWS). The results showed that SWS involves replay of the firing patterns found during activity, with the ordering of the firing retained during sleep. Norman *et al*. (2005) elaborated on a potential role for sleep in the CLS model. They argued that sleep provides an opportunity for both hippocampal replay, and also for restructuring/strengthening memories via an oscillating learning algorithm (Norman *et al*. 2006). These offline learning processes during sleep are argued to play a critical role in training neocortical systems—indeed, separate ‘wake’ and ‘sleep’ phases are central to neural network learning algorithms that include biologically plausible forms of error-driven training (O'Reilly 1996).

In the last 10 years a wealth of further neural evidence has amassed in support of some link between sleep and consolidation of hippocampal memories (e.g. Cantero *et al*. 2003; Buzsaki 2005; Dragoi & Buzsaki 2006). These advances have been paralleled in behavioural data, although this area remains controversial because of the many potential confounds involved in sleep research. For example, a study that shows superior performance on a memory task after overnight sleep as opposed to an equivalent time awake during the day may be confounded with time-of-day effects on performance (Keisler *et al*. 2007). If, on the other hand, time of day effects are controlled for by comparing overnight sleep with sleep deprivation overnight, then other confounds are possible relating to the effects of sleep deprivation. Several recent reviews cover this debate in detail (e.g. Vertes 2004; Stickgold & Walker 2005; Vertes & Siegel 2005), with recent methodological advances (reviewed by Walker 2005) providing a convincing case in favour of a significant (if not unique) contribution of sleep to improvements in memory performance.

The declarative/non-declarative distinction has been useful in evaluating the influence of sleep on memory. It is relatively uncontroversial that aspects of procedural and perceptual (non-declarative) performance can improve following sleep. For example, Karni *et al*. (1994) used selective sleep deprivation to show that visual texture discrimination was benefited by REM sleep but not by SWS. Similarly, Fenn *et al*. (2003) demonstrated a sleep-associated improvement in the ability to interpret synthesized speech. If listeners practised the task in the morning, the passing of time awake would lead to deterioration in perceptual skill by the evening. Thus, the change overnight could be thought of as a recovery of the originally strong performance (cf. Brawn *et al*. 2008). Other studies found similar performance enhancements across a range of non-declarative tasks (see Walker 2005; Born *et al*. 2006).

Until recently, the situation for declarative memory was less clear-cut, with a mixture of positive and negative results roughly balancing each other out (cf. Stickgold & Walker 2005). However, recent studies show more robust effects of sleep, at least for some types of declarative memory. Plihal & Born (1997) once again used selective deprivation to show an effect on paired-associate recall, although in this case the benefit was found for participants who were allowed SWS. Gais *et al*. (2002) went further, demonstrating that paired-associate learning prior to sleep influenced neural activity during sleep. Specifically, EEG measurements of sleep spindle activity (short bursts of higher frequency waves) showed a rise associated with declarative learning in the non-REM component of early sleep. Marshall *et al*. (2006) provided an even clearer link between non-REM sleep and declarative memory by showing that artificially enhancing the oscillations in SWS using transcranial oscillating potentials led to improved retention of paired associates following sleep (see also Ellenbogen *et al*. 2006; Drosopoulos *et al*. 2007).

These and other studies have led to the conclusion that sleep benefits the consolidation of both declarative and non-declarative memories, with REM sleep implicated in the case of procedural and perceptual abilities and SWS involved in the consolidation of declarative memory. However, this dichotomy may be too simplistic, and it is important to note that learning may well involve both types of memory, either independently or interactively. Some recent studies have demonstrated that sleep can help to alter the form of memories, leading to new insights (Wagner *et al*. 2004; Fisher *et al*. 2006), possibly through greater linkage between hippocampal and neocortical systems. Similarly, Ellenbogen *et al*. (2007) argue that sleep offers a means of integrating new information. In their case sleep facilitated the transitive inference to link pairs of premises. For example, given the information that B > C, C > D and D > E separately, participants found it easier to decide that B > E after sleep. This integrative aspect of sleep-related consolidation is a crucial one in the case of vocabulary, given that we need to be able to distinguish words from their neighbours (semantically, orthographically and phonologically) in order to recognize and understand them (cf. Dumay & Gaskell 2005, 2007).

## 2. Applying the cls model to spoken word learning

Based on the above review, we can outline how word learning might operate if it makes use of CLS principles. We begin by specifying the functional and anatomical organization of the neocortical networks involved in recognising spoken words. A particular focus here is a computational account of the perception and identification of spoken words constructed using a distributed connectionist model (the Distributed Cohort Model, Gaskell & Marslen-Wilson 1997, 1999). This network captures important functional properties of spoken word recognition, including optimally efficient use of incoming information in the speech signal, robustness to noise and variation in the perceptual form of speech, and competition between phonologically similar lexical items. All these forms of behaviour arise as emergent properties of a neural network model in which multiple, similar words are stored in overlapping neural representations. We therefore propose this model as an approximation to the neocortical component of the CLS account of word learning. In this section we begin by laying out the structure of the model, and the parallels between this neural network account and the anatomical organization of the cortical networks involved in perceiving speech. We then move onto a discussion of how CLS principles can be incorporated to provide an account of word learning.

### (a) The distributed cohort model and neocortical networks for speech perception

**Figure 1.**
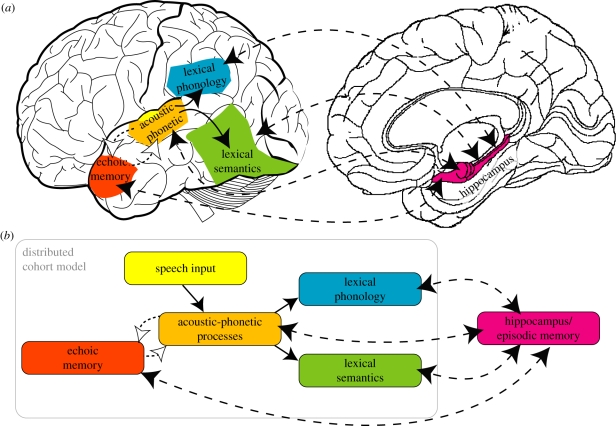
Neural and functional organization of systems involved in representing and learning spoken words. (*a*) Left temporal lobe regions involved in perceiving and comprehending spoken words (based on Hickok & Poeppel 2004; Davis & Johnsrude 2007) and their interactions with medial temporal systems for word learning. (*b*) Functional organization of the Distributed Cohort Model (Gaskell & Marslen-Wilson 1997, 1999; depicted within the grey box) with additional connections to hippocampal/episodic memory system for learning new words. In both diagrams, rapid cortico-cortico connections are shown with solid lines, and slower, cortico-hippocampal connections are shown with broken lines. Dotted lines with open arrow-heads show recurrent connections involved in maintaining acoustic-phonetic representations in echoic memory.

The Distributed Cohort Model (Gaskell & Marslen-Wilson 1997) uses a simple recurrent network (Elman 1990) to map from a sequence of acoustic–phonetic features representing ongoing speech input onto a distributed representation of lexical knowledge. While the input provided in simulations using the Distributed Cohort Model is abstracted from the surface detail of speech for computational convenience, we can assume that the input to the system corresponds to complex spectro-temporal feature representations encoded in primary auditory regions on the superior temporal plane (e.g. Patterson *et al*. 1995; Chi *et al*. 2005). From these features, both the model and current neuroscientific accounts postulate hierarchically organized processing pathways that extract different forms of abstract linguistic representation from ongoing speech input (see figure 1 for a depiction of the Distributed Cohort Model and corresponding regions of the temporal lobe).

In mapping sequences of speech segments onto lexical representations, the Distributed Cohort Model requires short-term storage so that information in the speech input can be accumulated over time, and sequences of phonemes can be discriminated. This is achieved in network simulations by including recurrent connections at the hidden unit level such that the model can take into account both the current input, and a representation of prior input during perception. These recurrent connections provided sufficient short-term storage for effective lexical processing in simulations thus far. However, in order to recognize temporally extended sequences, it might be that more complex computational architectures in which there is greater duplication of representations over time or a hierarchy of perceptual representations with progressively longer temporal receptive fields would be required. For this reason, we suggest that the hidden units, along with their recurrent connections, correspond to neuroanatomical systems that provide a form of transient memory for previously heard auditory input—auditory echoic memory functions subserved by anterior regions of the superior temporal gyrus (Buchsbaum *et al*. 2005; Price *et al*. 2005; Davis & Johnsrude 2007). The short-term storage provided by auditory echoic memory plays a particular role in the perception of spoken sentences (Mattys 1997) hence the frequent observation of anterior temporal activation in brain imaging studies of sentence comprehension (Scott *et al*. 2000; Davis & Johnsrude 2003; Humphries *et al*. 2005).

One distinctive property of the lexical representations that are the target output for the Distributed Cohort Model is that they separate the phonological form and the meaning of spoken words. These dual-outlets have distinct roles in accounting for the specific psycholinguistic tasks that are to be simulated by the model. The semantic output is more critical for making lexical/semantic decisions, producing semantic priming and for simulating tasks that involve access to word meaning. In contrast, the phonological output is more critical for making phonological decisions, repeating spoken words and non-words and in simulations of cross-modal repetition priming (Gaskell & Marslen-Wilson 1997, 1999). This separation of lexical identification into two distinct processes has clear parallels in current neuroanatomical accounts of auditory language pathways, which postulate distinct dorsal and ventral processing pathways (Hickok & Poeppel 2004, 2007; Davis & Johnsrude 2007). Several authors have converged on the suggestion that the critical function of the posterior-going auditory stream in the superior temporal gyrus and inferior parietal lobe is to map heard speech onto phonological representations involved in speaking (see Scott & Johnsrude 2003; Hickok & Poeppel 2004, 2007 for review and discussion).

In contrast to the neural substrates of phonological processing, there is rather less agreement concerning the critical pathways involved in accessing meaning from speech. This may reflect the fact that the neural systems involved in representing meaning are anatomically distributed and perhaps include regions that encode sensory-motor attributes of spoken word meaning (Barsalou 1999; Pulvermuller 1999; Hauk *et al*. 2004). However, at least for the majority of concrete, content nouns, we follow the proposal made by Hickok & Poeppel (2004, 2007) and others (Binder *et al*. 2000; Davis & Johnsrude 2007), and suggest that the posterior inferior temporal and fusiform gyri play a crucial role in accessing the meaning of spoken words. These regions therefore mostly clearly correspond to the semantic output of the Distributed Cohort Model.

### (b) Incorporating word learning into the distributed cohort model

In describing its architecture, and correspondences between different components with underlying neuroanatomical systems, we have omitted one critical feature of the model. That is we have not explained how appropriately weighted connections between units in the network are established. In simulations these connection weights are initialized to small random values and a backpropagation learning algorithm is used to adjust the weights in the network. This procedure allows gradual learning of a set of training words over the course of many presentations. The overlapping, distributed representations produced by this learning process permit accurate recognition of familiar words despite variable input and appropriate generalization to novel input sequences. One example of these properties is that following training with a large set of words, the model is able to generate an accurate phonological representation for non-words. However, in order to learn from presentation of these non-words (e.g. to activate an appropriate semantic representation or predict upcoming phonetic input) further changes to the connection weights of the network are required. As we have already described, neural network models trained using back-propagation show a dramatic form of interference when additional novel items are to be learned. That is, new words must be interleaved with existing words if they are to be acquired by the network.

We propose that a fully fledged CLS model of word learning needs a separate route in which sparse representations mediate between the representations of novel speech sequences (like the representations of sequences of speech features in the superior temporal gyrus) and lexical knowledge of the phonological form and meaning of new words (figure 1*b*). We propose an account in which connections between networks for speech perception in the lateral temporal lobe and memory systems in the medial temporal lobe (particularly the hippocampus) play a critical role in the acquisition of new words. Specifically, we propose that sparse representations of new words are rapidly acquired by the hippocampus (based on inputs provided by the cortical network). These connections support the recognition of newly learned words while existing neo-cortical connections operate in parallel and continue to support the identification of pre-existing, known words (figure 1).

If a dual-process model such as this underlies word learning and word recognition, what effects would be predicted for the time course of learning? Accessing the meaning and phonological form of a novel word should be viable as soon as the word has been learned, assuming that this information is provided during the learning process. Retrieval in these circumstances relies on the hippocampal route, which can operate independently of the main speech perception system. However, these hippocampal representations can only be integrated into the main neocortical recognition system offline over a longer time period. We suggest that sleep provides a means of reinstantiating hippocampal memories for neocortical learning. Data from amnesics suggest that such integration could take years or even decades to complete, although recent demonstrations of sleep effects on memory indicate that faster initial changes are also possible. In particular, effects such as Ellenbogen *et al*. (2007) finding that acquiring transitive representations of ordered picture pairs requires overnight consolidation provide strong evidence in favour of the idea that one of the key roles for sleep is the *integration* of hippocampal memories. Therefore, we can expect changes in neural representation of novel words following sleep, with shifts in the balance between hippocampal and neocortical representations. These changes may be associated with facilitated recognition of the novel words or access to their meanings, and by increases in the extent to which novel words influence the recognition of existing words.

Although hippocampal learning means that new form–meaning mappings can be acquired swiftly, there may be computational consequences of the fact that the new mapping is kept separate from the existing mappings. In particular, there may be time-course differences in terms of the speed of access of newly learned and existing words, depending on how quickly the two routes operate. Gaskell & Marslen-Wilson (1999, 2001) argued that one advantage of a PDP-style architecture for spoken-word recognition was that the state of the output units of the network directly reflects the likelihoods of the lexical candidates. For example, given the partial speech input /kæptI/, the network output would be a ‘blend’ of the distributed representations of the two matching words *captain* and *captive*, with the similarity of the blend to each target representation being proportional to the frequency of the two words. This state of affairs can be thought of as optimally efficient in terms of its use of partial information in the speech signal.

However, once the hippocampal route is brought into play and additional competitors have been learned, there is the possibility of losing optimality. Imagine the case where a listener learned the new word /kæptIk/ (*captick*). This new competitor would initially be learned via the hippocampal route, which would allow the appropriate distributed representation to be activated on presentation of the full spoken word. However, the state of activation of the output units prior to the final phoneme (/kæptI/) is potentially compromised. The neocortical route will still reflect the relative likelihoods of the two pre-existing words that it has been trained on, and the hippocampal route will reflect the episodic representation of the new word. However, the isolation of the hippocampal route means that the relative probability of the new word cannot be properly incorporated into the weighted blend of *captain* and *captive*. The outcome in these circumstances therefore depends on the balance between the two routes. If the hippocampal route is weighted too highly then something similar to catastrophic interference occurs, in that learning new words (e.g. *captick*) may interfere with the ability to recognize existing words (e.g. *captain* or *captive*). On the other hand, if the hippocampal route is weighted too weakly, the ability to retrieve stored information about the novel word is lost. One solution may be to have some kind of prioritization, such that the neocortical route is dominant up to the point where that route fails to recognize a familiar item. After this point, hippocampal activations can be taken into account (possibly by inducing a time delay in the hippocampal mapping). Such a solution would allow novel spoken words to be recognized, but would mean that they do not influence the recognition of existing competitors until they have been incorporated into the neocortical route. It might also suggest that the hippocampal route would have to operate more slowly than a purely neocortical recognition process and consequently that consolidation should serve to speed-up recognition of recently learned words.

## 3. Behavioural evidence for complementary processes in word learning

As we have seen in the previous section, the CLS account makes specific predictions concerning the neuroanatomical substrates of word learning and the functional characteristics of these processes. In particular, the CLS account predicts changes in the time-course of recognition of both novel and pre-existing words as a consequence of new learning and offline consolidation. It is only once consolidated into neocortical representations that newly learned words (e.g. *captick*) should be recognized quickly and efficiently, and be able to compete with existing words like *captain*. In this section we will review the existing literature on the time-course of identification of newly learned words with the goal of assessing evidence for the CLS account.

Tracking of speech–contingent eye movements has proved to be a rich source of information about the time-course of spoken language processing across a variety of domains (e.g. Tanenhaus *et al*. 1995; Altmann & Kamide 1999; Dahan *et al*. 2001). These experiments make use of a visual scene with objects chosen to inform about the lexical hypotheses and predictions that are made during the online processing of speech. For example, Allopenna *et al*. (1998) used pictures of cohort pairs (e.g. *beaker* and *beetle*), plus rhyming competitors (e.g. *speaker*) and unrelated competitors (e.g. *carriage*) and measured the probability of fixating each picture as participants listened to target words (e.g. *beaker*). The plot of fixation probability against time for each of these target types proved to be a highly sensitive measure of lexical activation for the different types of competitor, leading to a better understanding of the strength of cohort members and rhyming words as competitors in spoken word recognition.

These experiments have often made use of novel words as a way of manipulating the parameters of interest (e.g. semantic properties, Revill *et al*. 2008). Magnuson *et al*. (2003) used artificial lexicons to create stimulus sets that mimicked the cohort and rhyme relationships described above (e.g. target /pibo/ with cohort /pibu/, rhyme /dibo/ and unrelated /tupa/ distractors). Participants learned these words over the course of two days by associating them with visually presented abstract shapes. Magnuson *et al*. showed that word-like competition could be measured for these sets using probability fixation curves. Furthermore, frequency effects from newly learned neighbours were apparent in fixations even when the competitor shape was not present. In terms of the CLS approach, we can assume that the novel words were largely encoded hippocampally on the first day of these experiments and then jointly coded after sleep. Interestingly, although differences in performance levels between the two days were not dramatic, two experiments failed to show any effect of the relative likelihood of different types of competitor (cohort versus rhyme or high versus low frequency) on Day 1, though these effects were evident on the second day. It may be that the extra training on Day 1 allowed these relative weightings to be established more clearly on Day 2. However, an alternative explanation suggested by the CLS account is that the hippocampal route is less able to differentiate between candidates on the basis of likelihood. This would fit with the idea that the hippocampus simply requires some threshold to be reached in order to learn a mapping, with a habituation response to subsequent presentations.

Magnuson *et al*.'s (2003) final experiment went further in testing whether fixation probabilities to novel words could be modulated by the competitor environment of the listener's pre-existing lexicon. Novel words varying in neighbourhood environment (defined on the basis of the pre-existing lexicon) were learned as in previous experiments over the course of two days. Despite target frequency effects emerging (at least on Day 2), there were only hints of an effect of neighbourhood, and only for low frequency words. Thus, the novel lexicon could be considered ‘functionally isolated from the native lexicon’ (Magnuson *et al*. p. 223). This result might suggest that the novel words were held separate from the pre-existing lexicon in the hippocampal mapping (even on the second day). Alternatively this result could be a kind of context effect. Given that the stimuli at test were exclusively novel words, it may be that the language system is able to eliminate pre-existing words from the competition process early on, meaning that the neighbourhood environment of the novel words is no longer relevant (cf. Magnuson *et al*. 2008).

**Figure 2.**
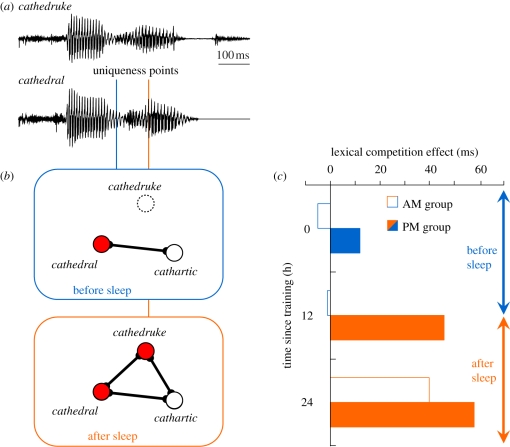
Impact of initial learning and sleep-associated consolidation on lexical representations and word recognition. (*a*) Speech waveforms for tokens of an existing word (*cathedral*) and a new word (*cathedruke*) with a marker showing the approximate time point at which the acoustic-phonetic input for *cathedral* diverges from all other known words (uniqueness point, cf. Marslen-Wilson 1984). The uniqueness point for *cathedral* is markedly later (orange line versus blue line) if the new word *cathedruke* must also be ruled out. (*b*) Lexical organization of these words after learning and before or after sleep-associated consolidation. Before sleep (blue box) the strongest lexical competitor for *cathedral* is *cathartic*, hence the uniqueness point is reached once this word can be ruled out (early uniqueness point shown in *a*). After sleep (orange box), the addition of a new lexical competitor is such that additional speech input is required to rule out *cathedruke* (late uniqueness point shown in *a*). (*c*) Pause detection response times showing the impact of learning and consolidation on lexical competition (data replotted from Dumay & Gaskell 2007). Two groups of participants were trained on novel words (e.g. *cathedruke*) at either 8.00 h or 20.00 h and tested on matched items with and without new lexical competitors 0, 12 or 24 h after training. Responses to existing words were significantly delayed by competition from newly learned words only for those conditions in which sleep intervened between training and testing (orange bars).

Rather than examining the effect of the existing lexicon on recognition of novel words, Gaskell & Dumay (2003) looked at whether learning novel words could influence the processing of existing words. The study made use of words such as *cathedral* that are uniquely identifiable early on in the word. Pseudowords that diverged from these existing words only at or after the uniqueness point (e.g. *cathedruke*) were selected to be taught to the participants as novel words. Critically, engagement of the novel words in lexical competition should lead to a delay in the recognition of words like *cathedral*, because the uniqueness point of the existing word had shifted closer to the end of the word (figure 2*a*). Participants were taught the novel words via phoneme monitoring and effects of this learning were tested the following day, with further cycles of exposure and test over the course of 5 days. When tested on the recognition of the form of the novel word using a two-alternative forced choice test with a minimally diverging foil (e.g. *cathedruce*), performance was close to ceiling at first test (93%), and remained at or above this level at later tests. However, the effect of novel word learning on recognition of the existing words was slow to emerge. These changes were measured using a lexical decision task measuring responses to *cathedral* in comparison with counterbalanced control words for which no neighbouring novel word was learned. A lexical competition effect was absent on days 2 and 3 but then emerged on the final two days. Furthermore, the effect was selective for the case where novel words were onset-matching neighbours of the existing word. As predicted by models of spoken word recognition such as the Distributed Cohort Model, equivalent overlap at the end but not the beginning of the word (e.g. *yothedral-cathedral*) showed no competition effect on any day.

Because the above experiment used multiple training sessions over several days it is not clear whether the lexical competition effect is simply dependent on a critical level of exposure being reached, or whether a consolidation period is also required. A further experiment addressed this issue using a single more extensive training session (36 presentations of each novel word) and lexical competition tests immediately and one week afterwards. These tests used a pause detection task, in which listeners are asked to monitor for silent pauses inserted in the existing words (e.g. *cathe_dral*). Mattys & Clark (2002) (see also Mattys *et al*. 2005) have demonstrated that this task is sensitive to the overall level of lexical activity at the pause position and hence should provide an index of lexical competition. Gaskell & Dumay (2003) showed that soon after the single intensive learning session there was excellent 2AFC recognition of the novel words, but no evidence of increased competition in pause detection for existing words. However, this competition effect was evident a week later on retest. The dissociation between form recognition and engagement in lexical competition is strong evidence in favour of the dual process CLS account of word learning, outlined above (see figure 2*b* for a sketch of these changes in lexical organization).

Subsequent studies have shown similar divisions between swift and delayed aspects of learning novel words. When novel words were assigned clear meanings in sentence contexts during training, the emergence of lexical competition was again delayed, though in this case competition effects were found a day after initial learning (Dumay *et al*. 2004). The same paper showed that the competition effect was not restricted to offset-diverging neighbours such as *cathedruke* and *cathedral*, but also for novel words that embedded the existing words (e.g. *shadowks* and *shadow*, cf. McQueen *et al.* 1994). This experiment also included both a recognition memory test and a free recall test, in which participants were given 3 min to recall as many of the novel items as they could. These explicit memory tests also showed improvements on the second day of the experiment. Thus delayed effects of lexical learning can also be detected in explicit memory tests of novel words with a sufficiently demanding task.

Given the delayed emergence of lexical competition and associated improvements in recall, Dumay & Gaskell (2007) attempted to tease apart effects of time and sleep after learning. Two groups of participants were trained on a set of novel words either at 08.00 or 20.00. Participants were then tested on their knowledge of these words straight after training, 12 and 24 h later. The tests included 2AFC recognition, free recall and pause detection, as in previous experiments. Both groups exhibited good recognition of the novel items immediately and at all retests, with no differences emerging. In contrast, pause detection showed an association between nocturnal sleep and the emergence of lexical competition effects. Test detection rates were slowed relative to control only for the conditions that had a night's sleep between training and test (i.e. after 12 and 24 h for the group starting in the evening, and after 24 h for the group starting in the morning). Because any circadian influences should apply equally to test and control conditions, these data are strong evidence in favour of an association between nocturnal sleep and integration of lexical information, and fit well with other studies implying an integrative role for sleep. Participants' free recall also showed an interaction between training group and time. There were improvements in free recall rate between subsequent test points in all cases where sleep intervened between the testing points and also when both test points followed sleep. The only case where improvement was not seen was for the group trained in the morning and then tested prior to sleep, where there was a marginally significant deterioration in recall rate. This observation fits well with the wider memory research in showing a protective aspect of sleep after learning (e.g. Ellenbogen *et al*. 2006).

The lexical competition studies described above (and the similarly delayed emergence of competition effects in studies of visual word learning, Bowers *et al*. 2005) are consistent with a model in which the hippocampus provides an initial means of binding representations of novel words in the short term. Integration of novel words with existing words occurs over a longer period of time and is associated with nocturnal sleep. Results that implicate the first night after learning as critical for consolidation do not imply a dichotomous transfer of knowledge. However, in those studies that test for an effect of a single night of sleep, it does appear to produce observable changes in behaviour. A further crucial prediction of the CLS approach is that the competition effect that emerges in the days following first learning should remain robust over a longer time-course, and may even strengthen. Although competition effects have not been tracked over a time-course of years (cf. Salasoo *et al*. 1985), Tamminen & Gaskell (2008) did examine the profile of lexical competition effects for several months following learning. Competition effects as measured by lexical decision were still robust at the final test point eight months after training, with some suggestion of enhanced competition effects by the end of the experiment. Although training only occurred at the beginning of the experiment, each test point did provide limited reactivation of novel word representations. Nonetheless, there were long periods of up to 17 weeks without any testing, and no significant reductions in the strength of competition effects after these gaps. Thus, to the best of our knowledge, the effects of learning that are found in these experiments are robust in the longer term.

One feature of all the experiments described so far is that they used multiple test sessions, including a test for lexical competition after learning but prior to sleep. Conceivably, this test session could have had a causal role in the emergence of lexical competition after sleep (e.g. through reactivation/reconsolidation, Walker *et al*. 2003; Hupbach *et al*. 2007; or through sleep-associated consolidation of test tasks, Plihal & Born 1997; Huber *et al*. 2004). We might therefore predict that competition effects would not be found after sleep if the competition test was not administered prior to sleep. Davis *et al*. (2009) tested this by teaching people two sets of novel words, one the day before testing and one on the day of testing. There was no competition effect from words learned that day, but words learned the previous day did produce a competition effect and faster response times in a speeded repetition task. This result does not rule out involvement of reconsolidation or task-learning in the effects described previously, but it does imply that lexical competition effects can emerge in the absence of reconsolidation of the pre-existing word representation, or repetition of test tasks. The partial transfer of the novel memory from hippocampal to neocortical storage stands out as the most parsimonious explanation of this result.

Although the above studies show that some aspects of lexical processing rely on a late-emerging representation, there are other aspects of word learning that do not require consolidation. Snoeren *et al*. (2009) looked at the ability of novel words to influence phoneme judgements in the context of compensation for the effects of assimilation. Previous research (Gaskell & Marslen-Wilson 1998) has shown that listeners can use the following context of a segment to compensate for the change in place of articulation that often occurs in speech production. For example, in the sequence *freight bearer*, the final consonant of *freight* is often similar to /p/ in connected speech. In perception, listeners make use of the following context to compensate for this shift, but this contextual compensation occurs more for words than for non-words. Snoeren *et al*. found that newly learned words immediately affected the tendency to use context to compensate for assimilation, and once again the strength of the compensation was no different for a separate set of items learned the previous day. The only effect of a 24-h period of consolidation was to facilitate response times overall, supporting the CLS proposal that word recognition should be speeded-up by overnight consolidation.

Sedin & colleagues (Sedin & Gaskell 2005; Sedin *et al*. in preparation) examined influences of newly learned words on speech perception using artificial phonetic continua, testing the extent to which novel words could bias listeners' perceptions of ambiguous phonemes. Many studies have shown that listeners' judgements in these cases are biased towards responses consistent with a word as opposed to a non-word (e.g. with an ambiguous (d/t) in the context of (d/t)ask, listeners are more likely to choose /t/, consistent with the word task; Ganong 1980). Sedin found that novel words could produce a similar bias immediately after learning and this effect remained of equivalent strength the following week. The only hint of a consolidation effect was again in response times, where speed benefits were found only at the second test point.

Leach & Samuel (2007) asked a related question concerning the influence of newly acquired lexical items on speech perception. They made use of the observation that spoken words (but not non-words) can drive changes to phoneme-category boundaries. For instance, Norris *et al*. (2003) showed that hearing words in which one phoneme was consistently replaced by an ambiguous phoneme (e.g. replacing the /s/ in *peace*, *rinse* and *crease* with an ambiguous form, half way between /s/ and /f/) causes a shift in listeners' categorization of an artificial continuum involving that phoneme. Crucially, this compensatory adjustment to the category boundary only occurred when the ambiguous phoneme was embedded in words, as opposed to pseudowords. Leach and Samuel tested whether newly learned novel words could show the same top-down influence on phoneme perception. They found that non-words can show this lexical effect as long as they were assigned a meaning in some way during training. Strongest effects emerged when pictures were associated with the novel words, when training did not involve pronunciation, and effects seemed to strengthen over the course of 5 days. The typical result, however, was that although type of training was crucial, the amount of time since first exposure was not. This contrasts strongly with the effects of lexical competition, where type of training seems irrelevant and time is crucial.

The latter experiments show that learning a new form can influence judgements about the form of speech soon after learning, which could be interpreted as evidence for immediate neocortical learning of novel words. Returning to the model sketched out at the beginning of this section, however, we can see that both hippocampal and neocortical routes have links with the lexical phonology level, and so in both cases we should see biases in phonological processing and the ability to evoke longer-term shifts in the categorization of speech. If, as we have speculated, the indirect hippocampal route to phonological representations is relatively slow then the response time advantage enjoyed by the 24-h delay conditions for phoneme monitoring in Snoeren *et al*. (2009) and for delayed repetition in Davis *et al*. (2009), may be due to a partial shift in reliance away from the hippocampal route and towards the direct neocortical route.

In sum, we have seen that a CLS approach to vocabulary acquisition fits well with the picture we have from behavioural data. Most aspects of lexical processing are available straight after learning and according to the CLS account may therefore be hippocampally mediated. However, there is a small but important set of behavioural properties that emerge later after sleep and which are probably associated with neocortical representations. Engagement in lexical competition is the best studied of these, but there also seem to be advantages in ability to recall words, and swifter processing of the novel words (in speeded repetition and assimilated segment detection) once integration into the neocortical route has begun. The neural predictions, however, can only be tested indirectly in behavioural studies. We therefore turn to neuropsychological and neuroimaging data as a more direct test of the CLS account.

## 4. Medial temporal contributions to word learning

In this section we review neuroscientific evidence in support of the CLS proposal that rapid initial learning is dependent on medial temporal lobe systems including the hippocampus (cf. McClelland *et al*. 1995; O'Reilly & Norman 2002). We begin by reviewing evidence for new word learning in amnesic patients with medial temporal lobe lesions before assessing convergent evidence from functional imaging studies, primarily using fMRI.

### (a) Neuropsychological evidence

In §1 we reviewed the basic pattern of impairment associated with damage to the hippocampus and surrounding regions of the medial temporal lobe. Here, we focus on the question of whether amnesics are able to learn new items of vocabulary. Studies of the late patient HM showed significantly impaired acquisition of words that came into the language after his bilateral resection of the medial temporal lobe (Gabrieli *et al*. 1988), although forced-choice and cued testing procedures revealed above-chance performance (O'Kane *et al*. 2004). Such findings suggest that amnesic syndromes are accompanied by impaired post-lesion learning of new words. However, recent anatomical investigations showed that HM had a more extensive bilateral lesion encompassing the hippocampus and adjacent regions of perirhinal and entorhinal cortex (Corkin 2002). Further evidence is therefore required if we are to assess the unique contribution of the hippocampus to word learning.

Patients with lesions confined to the hippocampus appear to show less marked impairments in the acquisition of new words. One important source of evidence concerning more circumscribed medial temporal lesions comes from observations of developmental amnesia following anoxic episodes during birth (Vargha-Khadem *et al*. 1997). This aetiology often leads to bilateral damage confined largely to the hippocampus (Gadian *et al*. 2000). Despite severe impairments in episodic memory, these children can show performance within the normal range in assessments of language function including tests of word knowledge (Vargha-Khadem *et al*. 1997; Gadian *et al*. 2000). Although these observations suggest that longer-term word learning can occur in the absence of the hippocampus, it might still be the case that the hippocampus normally plays a role in the initial acquisition and maintenance of new words.

To explore the impact of hippocampal lesions on initial acquisition a number of authors have used laboratory tests of word and concept learning in patients with developmental or adult-acquired lesions of the hippocampus. The consensus from these studies is that while new learning is possible, it remains weak by comparison with control participants. For instance, Martins *et al*. (2006) showed that a developmental amnesic with bilateral hippocampal lesions had impaired acquisition of new names and concepts in laboratory situations. Although this patient had a severe impairment (perhaps due to damage to the surrounding entorhinal and perirhinal regions), a patient with amnesia due to mamillary body lesions showed a milder impairment of word and concept learning compared with controls. A single case study of Jon, a developmental amnesic with hypoxia-induced bilateral hippocampal damage also showed significantly slowed acquisition of new words and associated semantics under laboratory conditions (Gardiner *et al*. 2008). Though some learning was possible it required greater numbers of repetition over several days of training. Thus, these results suggest that intact word knowledge in developmental amnesia arises from the operation of slower (perhaps cortically based) learning processes, consistent with the CLS account.

One question that arises in considering evidence from these developmental populations, however, is whether preservation of learning following early-onset hippocampal lesions reflects cortical learning mechanisms that remain plastic during childhood but would be absent following late acquired lesions. It is therefore reassuring that studies using adult amnesics largely confirm the pattern shown in these developmental studies. For instance, Verfaellie *et al*. (2000), showed that two amnesic patients with medial temporal lesions had impaired (though non-zero) knowledge of words that came into the language after the date of their lesions. Interestingly, of the two patients tested, a more severe impairment was observed in patient SS who subsequent to Herpes Simplex Encephalitis had lesions of both the hippocampus and adjacent entorhinal/perirhinal cortices. This is consistent with the profile of the developmental amnesic tested by Martins *et al*. (2006). Nonetheless, patient PS whose lesion was confined to the hippocampus also showed impaired knowledge of words that entered into the language after the anoxic episode that led to her amnesia. Two patients tested by Bayley and colleagues with more extensive lesions of the medial temporal lobe (encompassing most all of the hippocampus and rhinal cortices) also showed some post-lesion word learning since these patients could distinguish new words (e.g. *prozac* from foils *flozam*, *flozac*, etc) with above chance accuracy (Bayley *et al*. 2008). As well as word-form familiarity, both patients provided evidence of semantic knowledge of these words (e.g. knowing that a ‘website’ was a word associated with computers). Nonetheless, both patients had highly impoverished knowledge of new words by comparison with controls. Thus, evidence from adult and developmental amnesia would together imply that new word learning is severely impaired by bilateral hippocampal lesions. We therefore conclude that the initial acquisition of new words involves a similar dependence on medial temporal lobe learning systems to other forms of item-specific and associative learning (see Gooding *et al*. 2000 for a review). Some limited, residual learning abilities remain in amnesic patients, which we propose are supported by slower neocortical learning.

### (b) Functional neuroimaging evidence

Convergent evidence for hippocampal contributions to word learning has come from functional imaging studies in which participants are scanned while learning new words. One of the most persuasive demonstrations that hippocampal activation is associated with initial acquisition compared neural responses to consistent and inconsistent combinations of spoken pseudowords and pictures over five repetitions of these pairings (Breitenstein *et al*. 2005). Consistent pairings enabled participants to learn the picture–pseudoword associations, as shown by behavioural performance during and after scanning. Although activation differences between the consistent and inconsistent pairings were only observed in the right inferior frontal gyrus, there was also a significant linear decline in left hippocampal activity over five presentations of each consistent pairing, whereas the inconsistent pairings showed no such decline. Furthermore, a cross-subject analysis showed that participants who produced a greater response to the initial presentation of consistent pairings and a smaller decline in activation subsequently had better memory for the word–picture pairings. Thus, the authors concluded that hippocampal activation is associated with successful learning of spoken-word to picture pairings.

A subsequent fMRI experiment replicated this association between medial temporal activity and initial learning of form-meaning pairings for novel written words (Mestres-Misse *et al*. 2008). Mestres-Misse *et al*. contrasted fMRI responses to written pseudowords presented at the offset of sentences that provided either a consistent or inconsistent meaning for that pseudoword. Comparison of consistent and inconsistent meaning pairings revealed activation clusters in the precuneus, left thalamus, and anterior parahippocampal gyrus—the latter cluster probably including perirhinal regions lesioned in patients with more severe impairments of word learning. Interestingly, an ERP version of this experiment showed a progressive reduction in the magnitude of the N400 to sentence-final non-words provided with consistent meanings (Mestres-Misse *et al*. 2007). Evidence from intracranial ERPs might suggest that at least one plausible generator of the N400 effect is localized in medial temporal regions consistent with the activation observed in their fMRI paradigm (Nobre *et al*. 1994; McCarthy *et al*. 1995), though this localization has been disputed (see Lau *et al*. 2008). These findings, like the results of Breitenstein, suggest a role for the medial temporal lobe in learning associations between pseudowords and meanings. However, the studies leave unclear whether activation reflects involvement of medial temporal lobe systems in associative learning (consistent with hippocampal activation during successful learning of word–word and face–name associations: Henke *et al*. 1997; Sperling *et al*. 2003; Prince *et al*. 2005), or a more general role in initial encoding of novel stimuli (Strange *et al*. 1999; Yamaguchi *et al*. 2004).

Two recent studies in fMRI and PET, respectively, answer this question; first by showing that hippocampal activation is associated with successful encoding of novel spoken pseudowords in the absence of accompanying pictures or semantic information (Davis *et al*. 2009), second by showing equivalent medial temporal activity for form learning and associative learning (Paulesu *et al*. 2009). In Davis *et al*. (which will be reviewed in more detail subsequently), participants were presented with three sets of novel spoken pseudowords of which two had received extensive training prior to scanning. Activation of the hippocampus was primarily observed for those pseudowords that were entirely novel at the time of presentation, and declined rapidly with subsequent repetitions when these items were no longer truly novel. Furthermore, the magnitude of initial activation to novel pseudowords and the magnitude of the subsequent decline in activity on repetition predicted performance in a post-scanning forced-choice recognition memory test. Hence, this study suggests a role for the hippocampus in initial encoding of novel word forms, irrespective of whether participants associate these items with pictures or other semantic information. Confirmation of similar medial temporal involvement in form learning and associative learning comes from Paulesu *et al*. (2009) who used PET to compare responses to words and pseudowords in both these tasks. Again, we will discuss cortical activation observed in this study subsequently, however, with regard to the medial temporal lobe both associative and form learning responses to pseudowords declined over time in the anterior parahippocampal gyrus and temporal pole. Form learning and associative learning thus recruit similar medial temporal structures—though in this study these were located outside of the hippocampus.

In all these studies medial temporal activation during word learning reduced rapidly with stimulus repetition and most clearly predicted behavioural measures of successful learning (though the Paulesu study did not test this correlation). Such findings support the CLS proposal that medial temporal lobe systems are specialized for the rapid acquisition of new information, including language. This account would also explain the absence of hippocampal activation in studies in which participants received extensive training on novel items prior to testing (Sandak *et al*. 2004; Majerus *et al*. 2005). We next turn to evidence for the second neural prediction of the CLS account: that neocortical representations support long-term recognition of words and that cortical representations of pseudowords emerge slowly following stimulus familiarization and offline consolidation.

## 5. Cortical contributions to word learning

In contrast to medial temporal lobe systems, which are proposed to play a time-limited role in initial acquisition of spoken words, the CLS account predicts that a common set of cortical systems are involved in acquiring stable representations of new words and retaining those representations over the longer term. In assessing the contribution of cortical regions to word learning, then, we will first consider evidence from neuropsychology concerning the neural regions that support knowledge of familiar words. In a second section, we report a meta-analysis of functional imaging studies which contrasted neural responses to familiar words and unfamiliar (i.e. untrained) pseudowords. This meta-analysis assists in the interpretation of functional imaging studies that assess changes to the neural response to spoken pseudowords during and after familiarization. A stringent test of these studies is whether they provide evidence concerning the time-course by which pseudoword responses become word-like during and subsequent to acquisition. The CLS account makes the strong prediction that offline consolidation is required to generate stable neocortical representations of pseudowords.

### (a) Neuropsychological evidence

An important source of evidence concerning the cortical systems that are critical for word recognition comes from neuropsychological studies assessing brain areas that produce a significant impairment in the comprehension of familiar words when lesioned. In reviewing this work, we will focus on studies that link behavioural assessment of groups of neuropsychological patients to data from MRI scans revealing the location and extent of individual patient's lesions. Voxel-by-voxel analysis methods similar to those employed in functional brain imaging can thus be used for statistical assessment of lesion–symptom associations. Interestingly, the results of these analyses provide two distinct answers to the question of which brain regions produce impaired single word comprehension when lesioned.

Lesion–symptom maps for speech comprehension impairments in a large group of aphasic stroke patients suggest an association between lesions and impaired comprehension in a region of the posterior middle temporal gyrus (MTG) extending into inferior temporal regions (Bates *et al*. 2003). This anatomical location overlaps substantially with the posterior temporal system highlighted as contributing to lexical semantic processing in figure 1*a*. Since Bates and co-workers used a single, composite measure of comprehension (derived from behavioural responses to ‘yes/no’ questions, following simple and complex commands, and an assessment of single word comprehension using word to picture/object matching), it is unclear whether comprehension impairments arise from impaired lexical processing. It is therefore encouraging that a follow-up study using a more sophisticated language battery (Dronkers *et al*. 2004) suggests that posterior MTG lesions are specifically associated with impairments of single word comprehension. Only posterior MTG lesions produced an impairment even for the simplest test sentences used, irrespective of syntactic complexity. A similar association between posterior inferior temporal lesions and impaired comprehension has also been reported by Peelle (Peelle *et al*. 2008) in a study of semantic dementia patients. Nonetheless, it remains unclear whether impairments are in word-form recognition or in the form-to-meaning mapping.

However, other studies that assess lesions associated with progressive comprehension impairments in semantic dementia patients (Mummery *et al*. 2000; Williams *et al*. 2005), or that use a range of aetiologies (Tyler *et al*. 2005*a*,*b*) would suggest a more anterior locus of the representations that support single word comprehension. For example, the speed with which patients respond in lexical decision is predicted by lesions in antero-lateral regions of the temporal lobe including the temporal pole (Tyler *et al*. 2005*a*), as is a measure of semantic priming (Tyler *et al*. 2005*b*). Further evidence for an association between damage to anterior temporal regions and deficits in the recognition of words and other familiar stimuli comes from studies of patients with multimodal semantic impairments following the temporal lobe variant of fronto-temporal dementia (semantic dementia). These patients suffer from a progressive, degenerative disorder that leads to significantly impaired semantic knowledge whether accessed from speech, writing or pictures and probed using forced choice, naming, drawing or generation tasks (Patterson *et al*. 2007). Voxel-based analysis methods have shown that cortical degeneration (Mummery *et al*. 2000; Williams *et al*. 2005) and hypometabolism (Nestor *et al*. 2006; Desgranges *et al*. 2007) in anterior temporal regions surrounding the temporal pole is most clearly associated with the degree of semantic impairment observed.

At present, it is unclear how we are to reconcile results suggesting that lesions to two distinct regions of the temporal lobe can both produce impairments of word recognition in patient populations. One plausible interpretation is that both anterior and posterior temporal regions contribute to successful recognition of familiar words in different ways. For example, Patterson, Rogers and others (Rogers *et al*. 2004; Patterson *et al*. 2007) suggest that the anterior temporal lobe forms a semantic hub that binds together neurally distributed representations of perceptual and conceptual features stored in more posterior regions. One speculative extension of this proposal in the context of the CLS account is to suggest that anterior temporal regions lesioned in semantic dementia provide input to the hippocampus. It might therefore be that anterior temporal lesions lead to a progressive decline in comprehension because damage impairs the ability to acquire new representations, and that these processes are also required if knowledge of familiar words is to be retained. Further investigation of word learning in these populations would be valuable.

### (b) Meta-analysis of word and pseudoword responses in brain imaging

Given this uncertainty concerning the role of anterior and posterior temporal lobe regions in the recognition of familiar words it is clear that functional imaging can provide important additional evidence concerning the cortical systems supporting word recognition. We therefore report a meta-analysis of published PET and fMRI studies that include a direct contrast between spoken words and pseudowords. We use an Activation Likelihood Estimation (ALE) method to derive a statistical map of the brain regions in which activation differences between words and pseudowords are expected (Turkeltaub *et al*. 2002). This procedure is implemented in software [GingerALE v1.1 from http://www.brainmap.org] that incorporates a false discovery rate (FDR) correction for multiple comparisons (Laird *et al*. 2005). We limited our coverage to studies that (i) report activation foci in a standard coordinate space (either the Talairach Atlas or the MNI152 average brain, see Brett *et al*. 2002) and (ii) conduct random-effects analyses of activation maps collected from groups of healthy adult participants.

We analysed all PET/fMRI studies of spoken word recognition that report the contrast between familiar words and phonologically well-formed, clearly spoken pseudowords in either direction. That is, we assessed regions involved in representing familiar items (responding more strongly to words) and regions that contribute to perception of pseudowords (responding more to pseudowords). This focus on the word/pseudoword contrast excludes studies in which non-word stimuli were physically distorted/degraded, or unintelligible versions of spoken words (e.g. Mummery *et al*. 1999), or non-linguistic stimuli (tones or similar, Binder *et al*. 2000). Since our goal was to assess neural representations of familiar words, rather than systems that are differentially engaged by task-specific processes for words and pseudowords, we also excluded studies in which the word/non-word contrast was confounded by task differences (such as the studies reviewed in Binder *et al*. 2000). Following these exclusions, 11 studies reporting a direct contrast between responses to spoken words and pseudowords remained, as summarized in table 1. Since certain of these studies included multiple contrasts, we focus our meta-analysis on main effects of lexicality, averaging over stimulus and task manipulations as appropriate. Where the results permitted (e.g. Majerus *et al*. 2005; Davis *et al*. 2009) we excluded conditions in which participants were exposed to certain pseudowords before scanning.

**Table 1.** Studies included in the meta-analysis. Those labelled asterisk (*) report peak activations for the Talairach & Tournaux (1988) brain atlas and have been transformed into the MNI152 average brain for analysis and reporting.

citation	imaging modality	number of participants	task	number of foci	
				word > pseudo	pseudo > word
Binder *et al*. (2000)*	fMRI	28	simple auditory detection (block onset)	3	0
Davis *et al*. (2009)	fMRI	16	pause-detection (non-target trials)	0	7
Gagnepain *et al*. (2008)	fMRI	18	lexical decision (filtered)	8	4
Kotz *et al*. (2002)	fMRI	13	lexical decision (paired priming)	7	6
Majerus *et al*. (2002)	PET	11	repetition/passive listening	7	1
Majerus *et al*. (2005)	PET	12	speeded repetition	2	3
Orfanidou *et al*. (2006)	fMRI	13	lexical decision (priming)	33	0
Prabhakaran *et al*. (2006)*	fMRI	15	lexical decision	0	1
Raettig & Kotz (2008)	fMRI	16	lexical decision	4	2
Sabri *et al*. (2008)*	fMRI	28	one-back detection (attend/not)	1	1
Xaio *et al*. (2005)*	fMRI	14	lexical decision	3	4
			**total**	68	29

All peak coordinates reported in the papers listed in table 1 were included in the meta-analysis. Those studies labelled with asterisks (*) in table 1 report activation foci in the space defined by the Talairach atlas and were converted into MNI space using the conversion routine supplied with the GingerALE software (see http://www.brainmap.org/icbm). In computing ALE maps for the word > pseudoword (w > p) and pseudoword > word (*p* > w) contrasts, we used a smoothing kernel with a full-width half maximum of 10 mm, and computed 5000 permutations in assessing the statistical significance of the resulting ALE map. Applying a statistical threshold of FDR *q* < 0.05 resulted in corrected statistical thresholds of *p* < 0.0038 for w > p and *p* < 0.0036 for *p* > w. Only clusters of voxels that exceeded a minimum cluster volume of 100 mm^3^ are reported.

**Figure 3.**
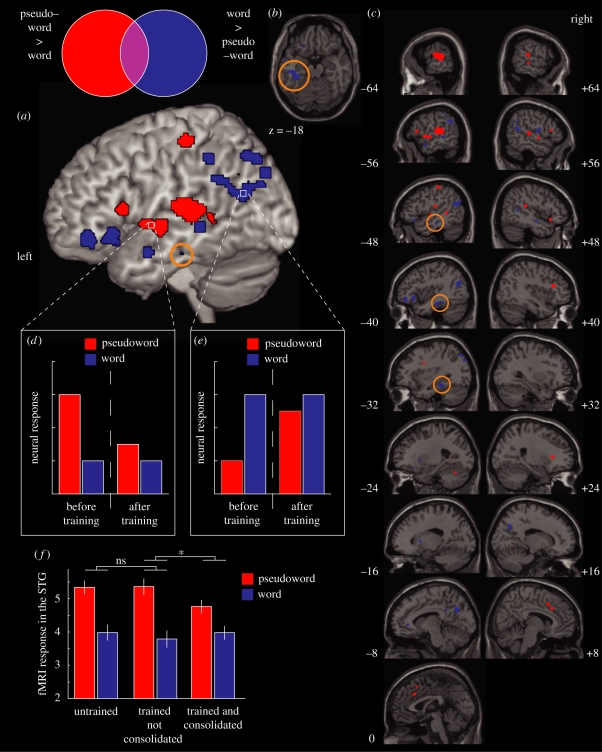
Activation Likelihood Estimation (ALE) map derived from 97 peak voxels from 11 functional imaging studies comparing neural responses to spoken words and pseudowords. ALE maps are thresholded at *p* < 0.05 FDR corrected, and only clusters larger than 100 mm^3^ are shown. Additional activation for pseudowords compared with words (red) and words compared with pseudowords (blue) is shown (*a*) rendered onto the left hemisphere, (*b*) displayed on an axial and (*c*) multiple sagittal slices of the MNI canonical brain (*z* and *x* coordinates as shown). Note inferior temporal and fusiform activation for word >pseudoword (circled in orange) is largely hidden in the rendering but apparent on the axial slice and sagittal slices *x* = −48, −40 and −32. (*d*,*e*) Response profiles showing predicted changes in neural responses owing to familarization with pseudowords: (*d*) Within regions that initially show an additional response to pseudowords (red in figure 3*a*–*c*, e.g. STG, posterior inferior frontal gyrus). For these regions we predict a diminished response to pseudowords after training. (*d*) Predicted response within regions that show an additional response to real words (anterior MTG, anterior fusiform, supramarginal gyrus, blue in figure 3*a*–*c*), we predict an increased response to pseudowords following training. (*f*) fMRI responses in a region of the STG overlapping with areas shown in red in figure 3*a*–*c* (replotted from Davis *et al*. 2009). An equivalent, additional response to pseudowords was seen for items that were untrained at the time of scanning or trained but not consolidated (i.e. learned on the same day as scanning). However, there was a significant lexicality by consolidation interaction with a reduced response to pseudowords that were trained and consolidated (i.e. learned on the day before scanning).

In describing the pattern of differential responses observed for words and pseudowords we will focus on responses in left hemisphere regions that as reviewed previously have been argued to be critical for the comprehension of spoken language (see §2 and figure 1*a*). We begin by describing the elevated responses observed for pseudowords shown in red in figure 3*a*–*c* and table 2. These provide putative neural correlates of phonological-encoding processes that are challenged during the initial processing of unfamiliar spoken items and that may therefore contribute to initial encoding of pseudowords in conjunction with medial temporal regions.

**Table 2.** Activation likelihood estimation results for 29 peak voxels in studies reporting a greater neural response to spoken pseudowords than words (shown in red in figure 2). Results thresholded at *p* < 0.05 FDR corrected, and with clusters greater than 100 mm^3^ reported. Entries shown in bold are cluster summary statistics (including centre of mass and volume), entries in plain type show local maxima.

			MNI coordinates		
location	volume (mm^3^)	*p* (uncorrected)	*x*	*y*	*z*
**left superior temporal gyrus (mid-posterior)**	**4080**		**−61**	**−28**	**10**
left superior temporal gyrus		0.015	−60	−34	10
left superior temporal gyrus		0.013	−64	−22	12
**right superior temporal gyrus**	**1416**		**62**	**−26**	**3**
right superior temporal gyrus		0.007	64	−28	14
right superior temporal gyrus		0.007	58	−26	6
right middle temporal gyrus		0.007	60	−28	−6
right middle temporal gyrus		0.007	68	−24	−2
**left superior temporal gyrus (mid-anterior)**	**1024**		**−56**	**−6**	**−1**
left superior temporal gyrus		0.007	−60	−8	−4
left superior temporal gyrus		0.007	−50	−10	0
left superior temporal gyrus		0.007	−56	2	0
**anterior cingulate**	**656**		**7**	**23**	**34**
right cingulate gyrus		0.007	12	22	34
right cingulate gyrus		0.007	2	24	34
**right insula**	**264**		**49**	**−32**	**18**
right insula		0.006	50	−32	18
**left inferior frontal gyrus (opercularis)**	**232**		**−54**	**18**	**10**
left inferior frontal gyrus		0.007	−54	18	10
**right middle frontal gyrus**	**232**		**40**	**26**	**19**
right middle frontal gyrus		0.006	40	26	18
**left middle temporal gyrus (posterior)**	**224**		**−48**	**−44**	**4**
left middle temporal gyrus		0.007	−48	−44	4
**right medial frontal gyrus**	**224**		**8**	**14**	**44**
right medial frontal gyrus		0.007	8	14	44
**right cerebellum**	**216**		**12**	**−56**	**−8**
right cerebellum		0.007	12	−56	−8
**right insula**	**216**		**25**	**25**	**7**
right insula		0.006	26	26	8
**left superior frontal gyrus**	**216**		**−3**	**18**	**50**
left superior frontal gyrus		0.007	−4	18	50
**left postcentral gyrus**	**216**		**−48**	**−26**	**58**
left postcentral gyrus		0.007	−48	−26	58
**right inferior frontal gyrus**	**208**		**50**	**20**	**−10**
right inferior frontal gyrus		0.007	50	20	−10
**left insula**	**208**		**−46**	**−20**	**18**
left insula		0.007	−46	−20	18
**right middle temporal gyrus**	**200**		**58**	**−6**	**−2**
right middle temporal gyrus		0.007	58	−6	−4
**right inferior frontal gyrus**	**192**		**28**	**6**	**26**
right inferior frontal gyrus		0.007	28	6	26
**left parahippocampal gyrus**	**184**		**−27**	**−52**	**−2**
left parahippocampal gyrus		0.006	−26	−52	−2
**left cerebellum**	**160**		**−22**	**−62**	**−26**
left cerebellum		0.007	−22	−62	−26
**right inferior frontal gyrus**	**160**		**54**	**22**	**13**
right inferior frontal gyrus		0.007	54	22	12
**left precentral gyrus**	**160**		**−34**	**4**	**29**
left precentral gyrus		0.006	−34	4	28
left precentral gyrus		0.006	−34	4	30

In the superior portion of the left temporal lobe we see two distinct clusters that show an additional response to spoken pseudowords compared with real words. These clusters are located posterior and anterior to Heschl's Gyrus (in planum polare and planum temporale, respectively) extending into adjacent regions of the superior temporal gyrus. These clusters fall squarely within the dorsal and ventral speech processing pathways suggested to radiate out of primary auditory regions (Scott & Johnsrude 2003; Hickok & Poeppel 2004, 2007; Davis & Johnsrude 2007). There is good agreement between these various accounts that the dorsal-going pathway, running into the posterior temporal lobe and on to inferior parietal regions serves to map heard speech onto inferior frontal regions involved in generating the phonological representations required for articulation. It is therefore of interest that we observe an elevated response to pseudowords in left inferior frontal (opercularis) and premotor regions that putatively form part of the articulatory network identified by Hickok & Poeppel (2004) and Scott & Johnsrude (2003). A number of other regions that have been associated with mapping heard speech onto motoric responses also appear in this meta-analysis include the insula (Dronkers 1996), supplementary motor area and cerebellum. The close proximity of the dorsal superior temporal cluster to primary auditory regions would suggest that this region contributes to sub-lexical processing of speech, consistent with their elevated response for pseudowords. Convergent evidence for this interpretation comes from functional imaging studies that ascribe phonological functions to these posterior temporal regions. For example, fMRI has shown that the magnitude of the posterior Superior Temporal Gyrus (STG) response is correlated with speech intelligibility yet sensitive to acoustically distinct forms of speech distortion (Davis & Johnsrude 2003). Well-controlled speech/non-speech comparisons also produce posterior STG responses (Mottonen *et al*. 2006; Uppenkamp *et al*. 2006; Heinrich *et al*. 2008) as do studies that contrast neural responses to phonological and acoustic changes to spoken syllables (Jacquemot *et al*. 2003; Liebenthal *et al*. 2005; Raizada & Poldrack 2007).

In considering the function and organization of the ventral pathway for speech processing, there is rather more disagreement between the different accounts reviewed here. Some authors (Scott & Johnsrude 2003; Scott 2005) have proposed that this anterior pathway in the superior temporal lobe regions contributes to identification of familiar spoken words, whereas others (Hickok & Poeppel 2004, 2007) have focused on posterior inferior temporal pathways as being critically involved in mapping heard speech on to lexical and semantic representations. While neither account makes specific predictions concerning neural responses to pseudowords, it is of interest that the elevated response to pseudowords in the anterior superior temporal lobe is clearly in front of primary auditory cortex in the superior temporal gyrus and planum polare. Our observation of an elevated response to pseudowords in these anterior auditory fields appears most clearly in line with accounts proposing that these regions contribute to echoic representations of speech (cf. Buchsbaum *et al*. 2005; Davis & Johnsrude 2007), although other authors have proposed a role for these regions in syntactic or combinatorial language processes (Friederici 2002; Humphries *et al*. 2006; Hickok & Poeppel 2007). Further data on the response of this portion of the anterior superior temporal gyrus to pseudowords would be valuable.

We now turn to the meta-analysis of brain regions showing an elevated response to real words compared with pseudowords, displayed in blue in figure 3*a*–*c*, and summarized in table 3. These regions include neural substrates for cognitive processes that are uniquely available for real words such as making contact with stored representations of familiar phonological forms and initial access to associated word meanings (though as is apparent in table 1, not all of the studies included in the meta-analysis used tasks that specifically required either lexical or semantic processing). In line with the dual-route accounts described earlier, we see additional activation for spoken words in temporal lobe regions extending both anterior and posterior from those activations that were reported for the pseudoword contrast. We see a large cluster in the anterior STG and MTG extending towards the temporal pole, as well as a large posterior temporal cluster extending from the posterior MTG/STG into the inferior parietal lobe including the supramarginal gyrus. The spatial organization of these anterior and posterior temporal activations is consistent with the proposal that activations for real words are at a higher level of abstraction along anterior and posterior speech-processing pathways than those seen in the reverse contrast. Such findings parallel the results of a recent fMRI study using visual words and pseudowords in which familiar lexical items evoked activation at ‘higher’ levels of the ventral visual processing stream (Vinckier *et al*. 2007). Similarly, we would suggest that the spatial position of these activations is consistent with a hierarchical account proposed on the basis of functional imaging data by (Davis & Johnsrude 2003, 2007). Preferential responses to pseudowords lead to focal activation of phonological processes, whereas real words lead to focal activation of higher level, lexical representations. One complexity of the present results is that additional activation for real words is observed in all three temporal lobe pathways shown in figure 1*a*. While we do not have sufficient space here to review evidence for dissociations among these systems, these findings illustrate that representations of familiar words are to be found in multiple cortical systems, consistent with computational accounts such as the distributed cohort model in which both phonological and semantic representations can be considered part of the lexical system (figure 1*b*).

**Table 3.** Activation likelihood estimation results for 68 peak voxels in studies reporting a greater neural response to spoken words than pseudowords (shown in blue in figure 2). Results thresholded at *p* < 0.05 FDR corrected, and with clusters greater than 100 mm^3^ reported. Entries shown in bold are cluster summary statistics (centre of mass and volume), entries in plain type show local maxima.

			MNI coordinates		
location	volume (mm^3^)	*p* (uncorrected)	*x*	*y*	*z*
**left temporal/parietal junction**	2488		**−47**	**−64**	**25**
left middle temporal gyrus		0.009	−40	−70	24
left supramarginal gyrus		0.008	−54	−52	30
left superior temporal gyrus		0.007	−50	−58	22
left superior occipital gyrus		0.007	−44	−78	28
**left fusiform gyrus**	1320		**−38**	**−28**	**−18**
left inferior temporal gyrus		0.008	−40	−24	−18
left fusiform gyrus		0.008	−36	−34	−16
**right temporal/parietal junction**	1152		**53**	**−49**	**17**
right superior temporal gyrus		0.009	54	−48	14
right supramarginal gyrus		0.007	54	−50	30
**left inferior frontal gyrus (orbitalis)**	**792**		**−40**	**27**	**−9**
left inferior frontal gyrus		0.008	−38	26	−8
left inferior frontal gyrus		0.007	−48	24	−12
**left cuneus**	664		**−6**	**−66**	**32**
left cuneus		0.01	−6	−66	32
**right precuneus**	648		**14**	**−63**	**36**
right precuneus		0.007	14	−66	40
right precuneus		0.007	16	−66	30
right precuneus		0.007	12	−56	38
**left middle frontal gyrus**	632		**−39**	**43**	**−10**
left middle frontal gyrus		0.009	−38	42	−10
**left precuneus**	440		**−11**	**−46**	**29**
left precuneus		0.007	−12	−48	28
left cingulate gyrus		0.007	−10	−44	30
**right precentral gyrus**	416		**55**	**−5**	**13**
right precentral gyrus		0.008	56	−6	12
right precentral gyrus		0.008	54	−4	14
**left superior parietal**	328		**−33**	**−72**	**47**
left superior parietal lobule		0.007	−30	−76	46
left superior parietal lobule		0.007	−36	−68	48
**left putamen**	280		**−19**	**12**	**3**
left putamen		0.007	−22	12	2
left putamen		0.007	−16	12	4
**left precuneus**	144		**−32**	**−82**	**36**
left precuneus		0.007	−32	−82	36
**left middle temporal gyrus (anterior)**	136		**−54**	**0**	**−20**
left middle temporal gyrus		0.007	−56	0	−20
**right middle temporal gyrus**	128		**50**	**−9**	**−14**
right middle temporal gyrus		0.007	50	−8	−14
**left anterior cingulate**	128		**−8**	**38**	**0**
left anterior cingulate		0.007	−8	38	0
**left inferior parietal**	128		**−56**	**−42**	**46**
left inferior parietal lobule		0.007	−58	−42	46
**left middle frontal gyrus**	120		**−22**	**26**	**−14**
left middle frontal gyrus		0.007	−22	26	−16
**left middle temporal gyrus (posterior)**	120		**−56**	**−36**	**−2**
left middle temporal gyrus		0.007	−56	−36	−2
**left precuneus**	104		**−36**	**−62**	**32**
left precuneus		0.007	−36	−62	32

### (c) Imaging cortical correlates of word learning

The differential cortical responses to words and pseudowords reviewed above allow us to recast the critical experimental questions of word learning studies in neural terms. What learning processes are required to change neural response to pseudowords into responses that resemble real words? A number of studies have shown changes in the magnitude of neural responses to pseudowords following repeated presentation, and have linked these response changes to behavioural improvements and hence word learning. However, we would argue that response changes owing to learning should at least partially cancel response differences between pre-existing words and entirely novel pseudowords if we are to infer that cortical representations of newly learned items have become word-like.

For the neural response to a pseudoword to become fully word-like requires two opposite changes following familiarization: response decreases in regions that respond more to pseudowords, and response increases in regions that respond preferentially to familiar words. The brain regions that show these changes and the direction of observed effects can thus be predicted from that seen for comparisons of words and pseudowords on initial presentation. The two interaction profiles depicted in figure 3*d*,*e* therefore provide specific hypotheses concerning the cortical correlates of word learning. This section of the paper will review functional imaging studies that assess neural responses to pseudoword stimuli before, during and after familiarization with a view to testing for either of these two interactions. Building on behavioural evidence for offline consolidation, we predict that the emergence of robust, cortical representations of newly learned words requires not just initial familiarization, but also a period of offline consolidation. Evidence that these response changes can occur more rapidly has the potential to challenge the CLS account.

One study that tested for word-like changes to neural responses to pseudowords was reported by Orfanidou *et al*. (2006). They used event-related fMRI to measure neural responses during lexical decisions to spoken words and pseudowords, half of which were presented twice during scanning. Neural responses showed elevated responses to familiar words in many of the temporal lobe regions shown in the meta-analysis (figure 3). Critically, however, response differences between words and pseudowords were unaltered by stimulus repetition—neither of the interactions between stimulus repetition/familiarization and lexicality depicted in figure 3*d*,*e* were significant in the fMRI data. This was not because of the lack of any effect of stimulus repetition on neural responses. Response increases for repeated presentations were seen in dorsolateral and orbital prefrontal regions, lateral parietal regions and the precuneus. These activation increases partially overlapped with brain regions showing an increased response to familiar words (e.g. in the parietal lobe), however, these regions did not show the lexicality by repetition interaction depicted in figure 3*e* but rather words and pseudowords showed an equivalent response increased, consistent with memory processes recruited for both classes of items. Response reductions for repeated items were observed in bilateral inferior frontal regions, the SMA and posterior inferior temporal gyrus, though once more these were of equivalent magnitude for words and pseudowords (unlike the profile in figure 3*d*). Correlational analyses showed that reduced responses in inferior frontal and motor regions were correlated with the trial-by-trial magnitude of behavioural priming, suggesting that the expression of the repetition priming effect is linked with neural systems involved in decision-making and response execution (cf. Dobbins *et al*. 2004). Despite the presence of a lexicality by repetition interaction in behaviour, neural responses showed no evidence for the emergence of word-like representations for repeated pseudowords.

In contrast to the study reported by Orfanidou, other studies that explore how neural responses change as a result of familiarization with pseudowords have claimed cortical correlates of word learning. However, we must be careful to distinguish effects of familiarization from the task-based repetition effects that were documented by Orfanidou *et al*. (2006). Studies that only assess familiarization effects for pseudowords cannot differentiate between emerging cortical representations of newly learned words as distinct from effects of stimulus repetition. For instance, Breitenstein *et al*. (2005) report that neural responses in an anterior region of the fusiform showed a greater decline over five repetitions of consistent word-picture pairings than for inconsistent word-picture pairings. Our meta-analysis shows that this fusiform region ordinarily shows an increased response for words compared with pseudowords. Pseudoword training should have increased responses to trained items and hence the response reductions shown by Breitenstein make the neural response to familiarized pseudowords less, not more, word-like. Note that this observation does not negate any of the conclusions of the Breitenstein study concerning the contribution that the fusiform in conjunction with the hippocampus makes to the initial acquisition of word-picture associations.

Two other event-related fMRI studies have similarly shown response reductions to repetitions of spoken pseudowords (Graves *et al*. 2008; Rauschecker *et al*. 2008). Both studies show that multiple presentations of pseudowords led to a reduced neural response within brain regions shown in our meta-analysis to produce an elevated response to pseudowords (the STG, premotor cortex and SMA in Rauschecker *et al*. 2008; left STS, motor cortex and cerebellum in Graves *et al*. 2008). That both these studies show a reduced response in posterior superior temporal regions is encouraging since it suggests that the change in pseudoword responses is in the correct direction to reduce the response difference between pseudowords and words (the interaction depicted in figure 3*d*). However, neither study measured the effect of stimulus repetition for familiar words and so cannot detect interactions that provide evidence for word-like neural responses.

Critical for the interpretation of these event-related fMRI studies are the findings of two PET studies that did compare the effect of stimulus repetition on words and pseudowords (Majerus *et al*. 2005; Paulesu *et al*. 2009). Both studies show equivalent repetition suppression in the STG when spoken words and pseudowords are presented repeatedly prior to scanning (Majerus *et al*. 2005), or during scanning (Paulesu *et al*. 2009). Hence, in this study there is no lexicality by repetition interaction in either the posterior STG (Majerus *et al*. 2005) or the anterior STG (Paulesu *et al*. 2009). It therefore seems that had words been included in the fMRI studies of Rauschecker and Graves, then the required lexicality by repetition interaction would be absent and a main effect of repetition observed (cf. Orfanidou *et al*. 2006). One further finding from the Majerus *et al*. (2005) study is that low phonotactic frequency non-words did not show the same response decrease owing to familiarization as familiar words and high-phonotactic frequency non-words. This might suggest that cortical correlates of rapid response learning are limited to items that are either familiar or very similar to familiar words. Such a finding is consistent with the Distributed Cohort Model, in which the ability to generalize to novel pseudowords is determined by the degree of similarity to pre-existing words. It is precisely for these atypical, low-phonotactic frequency non-words that the model would struggle to generate an appropriate phonological representation, preventing cortical learning processes from producing short-term task-based repetition priming.

One fMRI study that both tested for a familiarity by repetition interaction and ruled out task repetition was presented by Gagnepain and colleagues (Gagnepain *et al*. 2008). In this event-related fMRI study, participants made lexical decisions to acoustically degraded spoken words and pseudowords of which 50 per cent had been presented previously for a phoneme decision task. Gagnepain observed a symmetrical interaction between lexicality and stimulus repetition with responses in left posterior STG and right peri-auditory areas, i.e. a reduced response for second presentations of words, and an enhanced response for second presentations of pseudowords. Thus, in the absence of a task-based explanation of response changes, repetition effects can differentiate words and pseudowords. Gagnepain and colleagues suggest that response increases for pseudowords reflect long-term encoding of previously unfamiliar items. However, these increases were observed in regions close to auditory cortex that showed an elevated response to pseudowords in our meta-analysis. This interaction serves to increase the neural response difference between pre-existing words and pseudowords, rather than decreasing this effect as predicted by figure 3*d*. Thus, the reactivation of recently acquired pseudoword representations has the opposite effect on measured neural activity to the difference between familiar words and unfamiliar pseudowords. This interaction profile is reminiscent of that observed in occipital and posterior fusiform regions in studies of neural repetition for written words (Fiebach *et al*. 2005) and faces (Henson *et al*. 2000, 2002), which similarly differentiate familiar and unfamiliar items. In each of these studies, repetition effects for unfamiliar items are either absent, or do not appropriately overlap with regions that show an elevated response to familiar items.

In summary, then, existing studies have failed to provide a convincing demonstration that repeated presentation of spoken pseudowords can lead to the emergence of word-like cortical responses over the time span of a typical functional imaging experiment. While it is difficult to argue from a null effect, particularly for a predicted interaction, it is striking that none of these PET and fMRI studies have satisfied the critical predictions depicted in figure 3*d*,*e* that interactions between lexicality and familiarization should overlap with response differences between words and pseudowords. The lack of a significant interaction, however, is directly predicted by the CLS account which proposes that short-term stimulus repetition alone is insufficient to produce stable, task-independent cortical representations of newly learnt pseudowords. Behavioural results reviewed previously in this paper would instead suggest that prior presentation and a sleep-associated consolidation process is necessary for newly learned words to have an equivalent lexical status to familiar words. Consequently, neural effects of a consolidation period that included sleep were assessed in an fMRI study reported by Davis *et al*. (2009).

Like certain other PET and fMRI studies reviewed previously, Davis and colleagues tested for interactions between prior familiarization and lexicality. Since two different tasks were used in training and testing (phoneme monitoring and gap-detection, respectively; Connine & Titone 1996; Mattys & Clark 2002), task-based response learning could not account for effects of training on neural responses. The experimental design used three matched sets of words and pseudowords, two of which were familiarized prior to scanning. One set of words and pseudowords was familiarized on the day before scanning (hence subject to overnight consolidation), another group of items was familiarized around 4 h before scanning (learned but not consolidated) and a third group presented only in the scanner (untrained controls). This experimental design allows a test of interactions that reflect the impact of initial learning alone and the additional effect of an overnight delay between training and testing on response differences between words and pseudowords. The design does not allow any effects found to be necessarily associated with sleep as opposed to an extended period of consolidation awake. However, given the behavioural data reviewed previously (Dumay & Gaskell 2007) and the fact that the same-day training also included ample time for awake consolidation (4 h), sleep is likely to be the principal driver of neocortical changes observed in the comparison of items learnt on the same or previous day to scanning. Comparison of untrained words and pseudowords also permitted an assessment of simple lexicality effects as shown in the meta-analysis. However, perhaps because the tasks used depend on processing the surface form of speech, elevated responses were observed for pseudowords compared with words and not vice versa. We will discuss in the concluding section the implications of the lack of an elevated, word-like response to pseudowords following training and consolidation.

For the untrained items, Davis *et al*. found an elevated response to pseudowords in the STG (anterior and posterior), and left cerebellum similar to those shown in figure 3 (results included in the meta-analysis). Consistent with the findings of Orfanidou *et al*. (2006), no interaction between same-day familiarization and lexicality was shown. That is, an overlapping set of regions showed an elevated response to pseudowords compared with words when these items had been extensively trained in the hours prior to scanning. Indeed, for this pseudoword versus word contrast, an elevated response to pseudowords occurred in a more extensive phonological network including a bilateral region of the motor cortex and the left SMA (though changes between untrained and trained items were not statistically significant). What is striking, however, is that the same comparison between pseudowords and words did not reach whole-brain corrected significance for items trained on the day prior to scanning. Indeed, we saw a significant consolidation by lexicality interaction in the STG, motor cortex and right cerebellum (figure 3*f*). As is apparent, the critical interaction was between words and pseudowords trained with overnight consolidation and items trained and tested on the same day (hence not subject to consolidation). Thus, if the criterion for the emergence of a stable lexical representation is a significant reduction in the elevated response to a trained pseudoword, then participants require both initial training and offline consolidation (in this case over 24 h between training and scanning) for learning to impact on cortical responses. This finding is entirely predicted by a CLS account in which cortical learning requires offline consolidation.

To be clear, this result does not imply that all neural correlates of word learning require overnight consolidation. We have already reviewed other results from the Davis *et al*. study showing that the degree to which participants become familiar with pseudowords that were presented for the first time during scanning is associated with the activation and subsequent decline in hippocampal responses (cf. Breitenstein *et al*. 2005). These data suggest that the role of the hippocampus in word learning appears confined to the initial acquisition of novel pseudowords, consistent with the CLS account. In the light of certain neuroimaging studies reviewed here (Majerus *et al*. 2005; Graves *et al*. 2008; Rauschecker *et al*. 2008) that show effects of repetition priming on neural responses to pseudowords, we must add a further significant caveat. These studies provide evidence for rapid neocortical learning that is involved in the acquisition and expression of stimulus-response associations (Schacter *et al*. 2004). This form of learning can produce significant facilitation of behavioural and cortical responses to spoken pseudowords—though only if they are similar to existing words (cf. Majerus *et al*. 2005). If response learning applies to spoken words and pseudowords, then these effects can be observed for both items (Orfanidou *et al*. 2006). However, differential repetition priming for words and pseudowords can more often be observed with greater priming for familiar words (Gagnepain *et al*. 2008). In these experiments, pseudoword priming effects are often in the wrong direction to create more word-like cortical responses (Gagnepain *et al*. 2008). We would contend, therefore, that rapid, response-based learning processes are limited to tuning of existing representations, and are (on their own) insufficient to establish cortical representations of new spoken words. In the CLS account there are limits to rapid cortical learning so as to avoid the catastrophic interference present in neural network simulations. Response-based learning processes cannot be invoked to explain the overnight consolidation effects that we have observed in our functional imaging study (Davis *et al*. 2009). Nor can task-based repetition priming effects explain the effect of overnight consolidation on the emergence of lexical competition in behavioural studies that were reviewed in §3. In all these studies, both the stimuli presented, and the responses measured differ between training and testing.

This separation of rapid stimulus-response learning (that can be achieved by cortical mechanisms without consolidation) and slower cortical learning produced by hippocampal encoding and offline consolidation has the potential to explain certain puzzling observations from the literature on word learning in amnesic patients. A number of studies suggest that newly acquired semantic knowledge in this population is to some extent rote-learned and inflexible. For instance, whereas control participants taught the phrase ‘venom-caused illness’ could retrieve the correct final word when cued by synonyms (e.g. ‘poison-caused…’, or ‘venom-induced…’) an amnesic patient EP failed to respond correctly unless the exact same words were used to cue the knowledge (Bayley & Squire 2002). Similarly, a developmental amnesic patient Jon could acquire new semantic/factual knowledge, but required many more repetitions than control participants (Gardiner *et al*. 2008). These data might suggest that isolated neocortical learning mechanisms responsible are limited to supporting stimulus-response association of whole forms. Hence, there may be important benefits that hippocampal learning and overnight consolidation provide in the non-damaged brain. This two-stage learning process supports flexible and generalizable knowledge more effectively than rote or piecemeal learning using neocortical systems alone.

## 6. Discussion and future directions

In this paper we have presented a cognitively and neuroanatomically informed account of word learning, grounded in principles that are well-established for learning other domains of knowledge. Our CLS account, while novel to the domain of word processing, can therefore draw on extensive empirical support for complementary hippocampal and neocortical learning systems in other domains, as well as evidence that sleep plays a specific role in the consolidation and unification of newly acquired neural representations in these two systems. Given the obvious debt that our CLS account owes to pre-existing accounts of memory formation, we will begin by making a few remarks concerning the value to memory theory more generally of evidence from the study of word learning. We will then consider some unanswered questions concerning the CLS account of word learning that should be pursued in further empirical research.

### (a) What can memory theory gain from considering word learning?

Word learning is a domain in which adult participants have a large and relatively uniform body of knowledge—there is a core vocabulary that is shared by all English speakers. Furthermore, the cognitive abilities and cortical processes that support our ability to recognize, understand and produce familiar words are (as this review illustrates) relatively well understood. Yet, monolingual adults continue to add to their vocabulary (e.g. ‘blog’), an ability that is also critical for successful second language learning. For these reasons, we suggest that word learning is an ideal subject area in which to explore interactions between systems that support pre-existing knowledge and new learning, in a domain that provides striking real-world application.

Word learning also provides clear examples of the multiple forms of knowledge that are critical for successful performance. Word representations are jointly perceptual (in systems involved in recognizing words), procedural (in motor systems for producing words) and semantic (in systems representing word meaning). Representations in each of these linked systems must be robust and able to generalize from individual experiences to novel input. For instance, we must recognize familiar words spoken by unfamiliar voices or establish the meaning of familiar words in unfamiliar contexts. These properties are ably demonstrated by neural network models (e.g. Gaskell & Marslen-Wilson 1997) that are subject to computational limitations typical of systems trained with distributed learning algorithms. The CLS account therefore proposes that the acquisition of distributed word representations should depend on medial temporal lobe systems that encode episodic representations of the form and meaning of novel words.

Empirical evidence concerning the nature of these interactions between episodic and perceptual/procedural memories of spoken words therefore provides a natural interpretation within CLS accounts. One point of view on complementary learning systems has considered perceptual/procedural and episodic learning to be distinct processes that operate on different types of information and that are probed using different sorts of test (e.g. Marshall & Born 2007). Rather, we would contend that most ecologically valid forms of learning exemplify more than one single type of knowledge, and are hence not embodied in any single system. Word learning provides examples of multiple forms of learning (perceptual, declarative and procedural) within a single domain and therefore provides a useful paradigm in which to assess dissociations and interactions among these different systems.

### (b) Unanswered questions for the CLS account of word learning

As the current review illustrates, existing evidence from neuropsychology and functional brain imaging concerning neural systems involved in word learning are largely consistent with the CLS account proposed here. However, more specific empirical tests of the predictions of the CLS account remain to be conducted. In particular, it should be noted that those behavioural data that most clearly show effects of overnight consolidation test knowledge of the form of spoken words and not their meaning (speeded repetition tasks, e.g. Davis *et al*. 2009; resolution of lexical competition, cf. Dumay & Gaskell 2007). We have argued that these data can be understood in terms of consolidation processes that achieve optimal, probabilistic processing of perceptual input and integration of new knowledge with existing representations. Although the role of consolidation in supporting efficient perceptual processing is relatively well understood, it is less clear whether other aspects of word learning (such as the acquisition of semantics) are also subject to overnight consolidation. As yet there is limited behavioural evidence concerning the long-term acquisition of representations of word meaning (e.g. McKay *et al*. 2008) and no empirical demonstration that semantic representations are subject to overnight consolidation. From a neuroscientific perspective we can ask whether all of the cortical systems that contribute to word knowledge (depicted in figures 1 and 3) show an equivalent dependence on overnight consolidation; or whether it is only representations of perceptual form (in superior temporal regions), and associated motor representations (in the precentral gyrus and right cerebellum) that show consolidation. The results of the fMRI study reported by Davis *et al*. (2009) would support this second conclusion but further empirical investigation would be helpful.

A further set of empirical questions concern the finding that recognition of lexical neighbours of newly learned words is altered by overnight, sleep-associated consolidation (Dumay & Gaskell 2007; figure 2). Although these results are striking, they fall short of showing a causal link between sleep and consolidation. Only by making direct interventions to sleep architecture in individual participants can we show that it is sleep itself (rather than some other state ordinarily associated with sleep) that is necessary for consolidation. With this result in hand, further experiments could then ask what features of sleep are necessary for consolidation. The existing literature on motor learning provides evidence to link consolidation to specific sleep stages (REM sleep, Plihal & Born 1997) and specific physiological processes (Huber *et al*. 2004). Conversely, declarative, episodic memories such as word-pair associations appear to be consolidated during stage 2 slow-wave sleep, presumably by the spindles and k-complexes that predominate in EEG signals recording during this sleep stage (Plihal & Born 1997; Marshall *et al*. 2006). Such findings naturally raise the question of which of these different sleep stages is associated with consolidation of word form knowledge, or indeed whether multiple sleep phases are required for consolidation of word representations that are both declarative and procedural (Dumay & Gaskell 2007).

Although we believe that existing brain imaging data on word learning are consistent with the CLS account, there has been only limited evidence for some of our most specific neural predictions. For instance, we reviewed evidence for hippocampal involvement during initial encoding (indexed by elevated fMRI activity), but so far there is little data to show how hippocampal and neocortical representations are coupled. Conversely, we have seen that overnight consolidation appears to reduce elevated cortical responses for pseudowords. However, we have not yet provided any evidence that either initial learning or overnight consolidation can produce an elevated response to pseudowords as a consequence of training. One possible explanation of this finding is that elevated responses to real words reflect neural correlates of semantic representations that were not part of the training procedure used in the study of Davis *et al*. (2009). Alternatively, it might be that more than a single day of training and overnight consolidation is required to increase the cortical response to new spoken pseudowords in areas that respond to familiar words. These and other questions are currently being addressed by follow-up functional imaging studies. A further set of questions concerns the nature of the neural connections that support consolidated representations of learned words. The CLS account predicts stronger connections within the cortical network and reduced connections with hippocampal representations for consolidated words. Further, more detailed evidence concerning the nature of neural interactions between neocortical and medial temporal systems at different stages during learning and at different time points after learning would therefore be a valuable test of the CLS account.

One final point to be addressed in future work concerns the relationship between neural systems that support short-term representations of pseudowords (such as in phonological working memory tasks, or that support repetition priming of pseudowords) and long-term acquisition. Behavioural evidence would suggest that phonological short-term memory (pSTM) capacity predicts vocabulary acquisition abilities both in children, and brain-injured adults (Baddeley *et al*. 1998). Imaging studies of auditory-verbal short-term memory have highlighted superior temporal and inferior parietal regions that contribute to echoic and rehearsal-based aspects of pSTM, respectively (Buchsbaum *et al*. 2005). However, the CLS account predicts that an additional consolidation process is required to turn these short-term representations into long-term lexical knowledge. Functional imaging tests of neural overlap between pSTM and long-term lexical learning would therefore provide an important bridge between the CLS account, and other, pre-existing data concerning the role of pSTM in word learning.

## 7. Conclusions

We have presented an integrative account of the acquisition and recognition of spoken words that combines CLS theory with the existing computational and neuroanatomical accounts of word recognition. A range of behavioural, neuropsychological and functional imaging evidence points towards differential contributions of medial temporal and neocortical systems to rapid initial acquisition and long-term consolidation of spoken words, respectively. Although this account remains under-explored in the domain of word learning there a number of parallels that we have highlighted between word learning and other memory domains. Thus, we believe that the CLS account can provide a firm foundation for cognitive and neuroscientific explorations of processes that are fundamental to language acquisition and processing in adults and infants alike.
